# Conversion to Mild Cognitive Impairment and Alzheimer’s Disease Dementia Related to Apathy, *APOE* Genotype and Antidepressant Use

**DOI:** 10.1177/08919887251335002

**Published:** 2025-04-14

**Authors:** Rubina Malik, Miguel Restrepo Martinez, Isis So, Elizabeth Finger, for the Alzheimer’s Disease Neuroimaging Initiative

**Affiliations:** 1 Department of Clinical Neurological Sciences, Schulich School of Medicine & Dentistry, 6221 Western University, London, ON, Canada; 2 Department of Psychiatry, 67593 Clinica Las Americas AUNA, Medellin, Colombia

**Keywords:** Alzheimer’s disease, apathy, antidepressant, *APOE*

## Abstract

**Objective:**

Apathy and *APOE* ε4 genotype are risk factors for developing Alzheimer’s disease dementia (ADD). Antidepressant use is known to induce apathy. This study aimed to examine associations between *APOE* ε4, apathy, and antidepressant use with progression from cognitively normal (CN) to mild cognitive impairments (MCI), and MCI to ADD.

**Methods:**

Participants aged 55-90 were recruited from the Alzheimer’s Disease Neuroimaging Initiative. Participants were CN or had MCI at baseline and had completed at least 3 consecutive study visits. The NPI and NPI-Q apathy subscales were used to index the presence of apathy. Antidepressants used by participants included SSRIs, SNRIs, and AYTADs. Cox proportional hazards analyses examined the combined effects of apathy, *APOE* ε4 genotype, and antidepressant use on conversion from CN to MCI and from MCI to ADD.

**Results:**

Apathy and *APOE* ε4 were associated with increased risk of conversion along the CN-MCI-ADD continuum. Antidepressant use was associated with progression from MCI to ADD, and progression from CN to MCI in non-apathetic *APOE* ε4 carriers.

**Conclusion:**

Our findings support apathy and *APOE* ε4 as robust predictors of conversion to MCI and ADD, and demonstrate novel associations between antidepressant use and conversion. Future research should explore whether antidepressant use in MCI and ADD causes apathetic symptoms or serves to index apathy/depression severity.

## Introduction

Apathy is the most common neuropsychiatric syndrome in Alzheimer’s disease (AD), occurring in an estimated 43% of patients with probable AD.^1^ Apathy is defined as diminished motivation for goal-directed behaviour and/or cognition.^2^ Common symptoms of apathy include reduced engagement in work, hobbies, social interactions, and personal hygiene practices. In patients with AD, apathy is associated with poor prognostic and functional outcomes, including accelerated cognitive decline and increased morbidity.^3,4^

Apathy is prevalent in the aging population as well, in those who are cognitively normal (CN) and those with mild cognitive impairment (MCI).^5^ The presence of apathy in older adults is a well-established marker of risk for progressing from CN to MCI, and MCI to AD.^5-7^ In individuals with MCI, apathy presents an approximate 54% increase in risk of conversion to dementia.^6^ A recent meta-analysis comprising over 20 000 participants demonstrated an approximate 2-fold increase in risk of conversion from CN to MCI in individuals with apathy.^8^ As such, apathy is a robust predictor of conversion. However, other factors, such as genetics and medication use, may modify the risk associated with apathy.

The *APOE* ε4 allele is the strongest known genetic risk factor for late-onset AD.^9^ Past research explored the convergence of neuropsychiatric symptoms in MCI, such as apathy, depression, and agitation, and *APOE* ε4 allele status; additive interactions between symptoms, including depression and apathy, and a positive *APOE* ε4 status were found to be associated with increased risk for developing dementia.^10^ However, the impact of apathy-*APOE* ε4 interactions, in isolation of other neuropsychiatric symptoms, on conversion from MCI to Alzheimer’s disease dementia (ADD) has not been examined. Genetic risks associated with conversion from CN to MCI are not well-known. A recent meta-analysis examined factors influencing conversion to MCI in CN individuals; results suggested apathy, independent of *APOE* ε4 status, predicted mild cognitive impairment.^8^ As such, the question of whether *APOE* ε4 status is a stable predictor of conversion along the CN-MCI-ADD continuum remains.

Antidepressant use is a mainstay treatment for depression in older adults; different classes of these drugs target certain neurotransmitters to modulate mood and behaviour.^11^ Common classes of antidepressants used to treat depression include selective serotonin reuptake inhibitors (SSRIs), serotonin norepinephrine reuptake inhibitors (SNRIs), and atypical antidepressants (AYTADs). Although antidepressants are helpful for managing most depressive symptoms, some studies have reported a link between antidepressants and risk of dementia. One study, for example, found over a 3-fold increase in risk of dementia in individuals aged 60 and over.^12^ However, others have found that antidepressants may have cognitive benefits; 1 study reports a nearly 20% reduced risk of dementia in 60-80-year-olds with depression.^13^

Prior studies on antidepressant use and conversion to dementia are limited by not considering apathy as a confounding factor. Indeed, symptoms of apathy can emerge soon after an antidepressant is started, giving rise to “antidepressant-induced apathy”.^14^ In a cohort of over 600 adult patients treated with an SSRI, SNRI or AYTAD, researchers found that nearly half the patients (46%) experienced emotional blunting, a key feature of apathy.^15^ In a retrospective study, 92% of older adults taking an SSRI experienced clinically significant apathy, independent of depressive symptoms.^13^ As such, the potential role of apathy in previous investigations of antidepressant use and risk of dementia cannot be ruled out.

Here, we aimed to characterize the effects of apathy, *APOE* genotype, and antidepressant use on risk of conversion from CN to MCI, and from MCI to ADD. We first examined possible links between *APOE* genotype and apathy on conversion. We expected to see increased risk of conversion to MCI and ADD in individuals with apathy and at least 1 *APOE ε*4 allele. Then, combined effects of apathy and antidepressant medication use on conversion rates were explored. We hypothesized that individuals taking an antidepressant and who endorse apathy would demonstrate heightened risk of conversion to MCI and ADD. Lastly, due to potential confounds of non-randomized distribution of antidepressant use across the *APOE* genotype groups, and possible non-random distribution of *APOE* genotypes across the antidepressant use groups, a sensitivity analysis examined the combined associations of *APOE* ε4, apathy, and antidepressant medication on conversion rates. We expected to see exacerbated effects, where possession of an *APOE* ε4 allele, endorsement of apathy, and antidepressant use would result in the greatest risk of conversion to MCI and ADD.

## Methods

### Participants

Data used in the preparation of this article were obtained from the Alzheimer’s Disease Neuroimaging Initiative (ADNI) database (adni.loni.usc.edu). The ADNI was launched in 2003 as a public-private partnership, led by Principal Investigator Michael W. Weiner, MD. The primary goal of ADNI has been to test whether serial magnetic resonance imaging (MRI), positron emission tomography (PET), other biological markers, and clinical and neuropsychological assessment can be combined to measure the progression of mild cognitive impairment (MCI) and early Alzheimer’s disease (AD). ADNI enrolls adults, ages 55–90 years, with mild cognitive impairment (MCI), Alzheimer’s disease dementia (ADD), and cognitively normal (CN) controls. Participants with MCI at baseline scored between 24–30 on the Mini Mental State Exam (MMSE), 0.5 on the Clinical Dementia Rating (CDR) scale, had objective memory loss, and had preserved cognitive abilities. Patients with ADD at baseline scored between 20–26 on the MMSE, 0.5 or 1 on the CDR, met clinical (NINCDS/ADRDA) criteria for probable AD, and had Wechsler Logical Memory II sub-scale scores consistent with ADD diagnosis (with specific cut off scores based on educational level). CN participants scored between 24–30 on the MMSE, 0 on the CDR, had preserved cognitive abilities and no objective memory loss, at baseline. Participants with pre-existing psychiatric illnesses, such as major depression disorders, were permitted for enrollment in ADNI’s CN group, provided that cognitive abilities were not affected by the illness. Data from participants enrolled in any of the ADNI1, ADNI2, ADNIGO, and ADNI3 phases, and who completed at least 3 study visits, and were CN or had MCI at baseline, were included in the current study. All participants provided written informed consent at enrollment as approved by local ethics committees.

### ADNI Measures

#### Neuropsychiatric Inventory (NPI)

The NPI and NPI-Questionnaire (NPIQ; a self-administered version of the NPI interview) are informant-based indices of neuropsychiatric symptoms exhibited over the previous month. The domains assessed by the NPI and NPIQ include hallucinations, delusions, agitation, depression, anxiety, elation/euphoria, apathy, disinhibition, irritability/lability, motor disturbance, sleep, and appetite. The presence of each symptom is recorded using a binary variable coding system, with 1 indicating presence, and 0 indicating absence.^16^

In the current study, the NPI and NPIQ were used to identify participants who did and did not endorse apathy throughout their enrollment in ADNI. Participants were binarily categorized into 2 groups: apathy present or apathy absent. For participants who converted to MCI or AD during the study, those who endorsed apathy prior to the conversion event were categorized as apathy present to examine the predictive force of apathy in diagnostic progression. Participants who did not convert during the study, but endorsed apathy at some point in their enrollment, were also categorized as apathy present.

#### Geriatric Depression Scale (GDS)

The GDS is a 15-item self-report measure of depressive symptoms in participants. Scores range from 0-30, with higher scores indicating elevated levels of depressive symptoms. Previous research demonstrates that 3 items on the GDS better represent symptoms of apathy rather than depression.^17^ These items include: “Have you dropped many of your activities and interests?”, “Do you prefer to stay at home, rather than going out and doing new things?”, and “Do you feel full of energy?” To parse out the relative contributions of apathy and depression to conversion, the current study used the GDS-12 score, calculated as the total GDS score excluding the 3 items related to apathy as discussed above.

#### Alzheimer’s Disease Assessment Scale Cognitive Subscale (ADAS-Cog)

The ADAS-Cog is a principal outcome measure in clinical trials for AD and is comprised of tasks used to investigate cognition in the following domains: word recall, commands, constructional praxis, naming, ideational praxis, orientation, word recognition, delayed recall, language comprehension and production, word-finding, concentration, and executive function. Total scores range from 0-70, with higher scores indicates more severe cognitive impairment.^18^ In this study, the ADAS-Cog was used to account for effects related to global cognitive impairment.

#### Clinical Dementia Rating Scale (CDR)

The CDR is a semi-structured interview of patients and their informants, conducted to evaluate dementia severity. The rating assesses the patient’s cognitive ability to function in 6 domains, including memory, orientation, judgment and problem solving, community affairs, home and hobbies and personal care. Each domain is assessed with 5-point scale: 0 = no impairment, 0.5 = questionable impairment, 1 = mild impairment, 2 = moderate impairment, and 3 = severe impairment. A global CDR score between 0-3 is calculated for each participant.^19^

#### *APOE* Genotype

*APOE* genotypes for participants were extracted from ADNI, which uses the Illumina method for genotyping.^20^ Participants were categorized based on *APOE* ε2 and *APOE* ε4 allele status, into 4 mutually exclusive groups: *APOE* ε4 carriers (defined as at least 1 ε4 allele and no ε2 allele, thus included ε34 and ε44), *APOE* ε2 carriers (defined as at least 1 ε2 allele and no ε4 allele, included ε23 and ε22 genotypes), genotype ε24, and genotype ε33. Participants heterozygous and homozygous for the ε2 and ε4 alleles were grouped in this way to increase statistical power. A small number of participants had an *APOE* ε24 genotype and were included in a sensitivity analysis.

#### Concurrent Medications

Concurrent medication logs were leveraged to identify participants prescribed antidepressant medications. Participants were assigned values of 1 (=yes) or 0 (=no) to indicate whether they were taking SSRIs, SNRIs, and/or AYTADs. Participants who were taking multiple categories of these medications were excluded from the analysis. SSRIs included citalopram, escitalopram, fluoxetine, paroxetine, and sertraline. SNRIs included duloxetine, desvenlafaxine, and venlafaxine. AYTADs included bupropion, mirtazapine, and trazadone. For the current study, participants were assigned a value of 1 or 0 to indicate antidepressant use (regardless of antidepressant class), or non-use, respectively, for every time interval at which medication data were made available in ADNI.

#### Diagnosis Change

Each participant in ADNI is provided a diagnosis at baseline when they enroll in the study. For each 6-month follow-up visit, an updated diagnosis status is indicated. In the current study, the visit at which a diagnostic conversion occurred (ie, CN to MCI or MCI to AD) was extracted.

### Statistical Analysis

Cox proportional-hazards (CPH) survival analyses were conducted using the Survival package in RStudio. The purpose of the analyses was to investigate the interactive effects of apathy, *APOE* genotype, and antidepressants on rate of diagnostic conversion (CN to MCI and MCI to AD). The *Event* variable was conversion, coded 1 for participants who convert, and 0 for censored participants. Participants who never converted throughout their ADNI visits were right-censored, because the period of observation expired prior to knowing whether the imperative conversion event would take place. The *Time* variable indicated months to conversion since study enrollment (baseline ADNI visit), or censoring time, defined as the last ADNI visit the participant completed, for participants who never converted.

**Figure 1.**
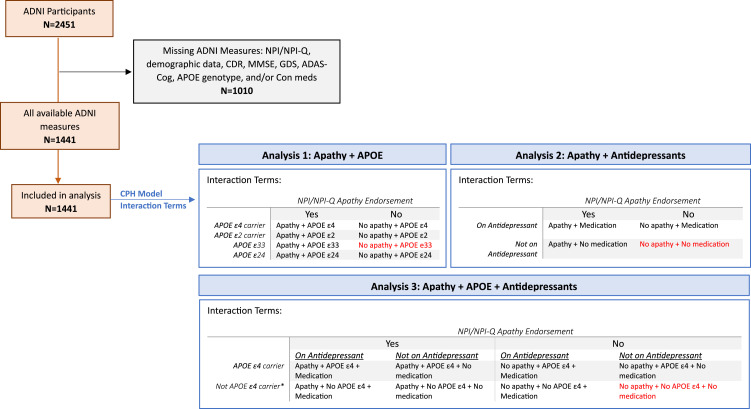
Screening ADNI participants and CPH analysis pipeline. Red = reference category. * Not *APOE* ε4 carrier = *APOE* ε2 carrier or *APOE* ε33.

To examine the additive effects of apathy, *APOE* genotype, and antidepressants, CPH models including terms for specific interaction effects were used (ie, apathy-*APOE* genotype interaction, apathy-antidepressant interaction, and apathy-*APOE*-antidepressant interaction). For the apathy-genotype analysis, for each genotype category participants were assigned to an Apathy or No Apathy group based on NPI/NPI-Q apathy endorsement, as depicted in Figure 1. These included: Apathy + *APOE* ε4, No apathy + *APOE* ε4, Apathy + *APOE* ε2, No apathy + *APOE* ε2, Apathy + *APOE* ε33, or No apathy + *APOE* ε33. No apathy + *APOE* ε33 served as the reference group. A sensitivity analysis with the inclusion of 2 additional interaction terms was conducted to account for possible confounding effects of *APOE* ε2ε4 heterozygosity: No apathy + *APOE* ε2ε4, Apathy + *APOE* ε2ε4.

For the apathy-antidepressant interaction analysis, each participant visit was assigned 1 of the following category terms, based on NPI/NPI-Q apathy endorsement and indication of antidepressant medication use at each available timepoint: Apathy + No medication, Apathy + Medication, No apathy + No medication, or No apathy + Medication. The No apathy + No medication group served as the reference category.

Lastly, for the apathy-*APOE*-antidepressant analysis, each participant visit was assigned to 1 of the following categories: Apathy + No medications + *APOE* ε4, Apathy + No medications + No *APOE* ε4, Apathy + Medications + *APOE* ε4, Apathy + Medications + No *APOE* ε4, No apathy + No medications + *APOE* ε4, No apathy + No medications + No *APOE* ε4, No apathy + Medications + *APOE* ε4, or No apathy + Medications + No *APOE* ε4. The No apathy + No medications + No *APOE* ε4 group was the reference category (Figure 1).

Time-dependent variables were extracted from each 6-month follow-up visit, up until conversion or censoring. The apathy-antidepressant and apathy-*APOE*-antidepressant interaction terms were treated as time-dependent variables. CDR global and ADAS-cog total scores were also included as time-dependent covariates to account for the effects of executive dysfunction and cognitive impairment on conversion. GDS-12 score was included as a time-dependent covariate to adjust for the relative effects of depression on conversion. Demographic covariates included age, sex (male = 0, female = 1), and years of education, with age being a time-dependent variable. Sensitivity analyses, with the exclusion of CDR global scores were conducted to account for any potential overlapping variance with diagnosis group categorizations.

Separate analyses were used to assess 2 conversion events of interest in 2 separate cohorts of participants: (1) conversion from CN to MCI, and (2) conversion from MCI to AD. The CN to MCI cohort analysis included participants who enrolled in the study as cognitively normal and remained so throughout the study, as well as those who converted from CN to MCI. The MCI to AD cohort analysis included participants who enrolled in the study with MCI and remained classified as MCI throughout the study or converted to AD. Participants who underwent 2 conversion events, from CN to MCI and MCI to AD, were excluded from the current study. The contribution of terms and covariates to the Cox proportional hazards models were assessed using omnibus likelihood ratio tests (LRT).

## Results

### Participants

A total of 1441 participants were eligible for the current study (Figure 1). Approximately 53% of participants were male, and the average age of participants was 73.3 years old. Eighteen percent of CN participants converted to MCI over the course of the study. Amongst these converters, 32% endorsed apathy prior to conversion, while 17% of matched non-converters endorsed apathy. Approximately 44% of participants converted from MCI to ADD over the course of the study. Within this group of MCI to ADD converters, over half of the participants (51%) endorsed apathy prior to conversion; in comparison, 74% of matched non-converters endorsed apathy.

Within both converter groups, there were no statistically significant differences in *APOE* genotype between those who did and did not endorse apathy. Within the CN, MCI, and CN to MCI groups, homozygous ε3 was the most common genotype, followed by ε4 carriers, with ε2 carriers comprising the smallest sample of participants in each group. In the MCI to ADD group, an *APOE* ε4 genotype was most common, followed by homozygous ε3. Additionally, a larger proportion of CN to MCI converters with apathy were taking SSRIs, SNRIs, or AYTADs, compared to those without apathy (Table 1).

**Table 1.** Kruskal-Wallis Analyses of Variance, Independent 2-Group Mann-Whitney U Tests, or Chi-Square Tests of Independence Were Used to Discern Group Differences for Relevant Study Variables.

	Total	CN stable	MCI stable	CN to MCI	MCI to ADD	Contrasts	CN to MCI + apathy	CN to MCI-apathy	Contrasts	MCI to ADD + apathy	MCI to ADD -apathy	Contrasts
N (with apathy)	1441	510 (90)	458 (337)	116	357	--	37	79	--	181	176	--
Age (SD)	73.33 (8.56)	71.68 (8.70)	72.80 (8.71)	76.72 (8.13)	75.24 (7.77)	*X*^2^(3) = 58.10, *P* < 0.001*	78.86 (7.61)	76.64 (8.25)	W = 197.5, *P* = 0.70	75.36 (7.54)	75.11 (8.02)	W = 1.57 × 10^4^, *P* = 0.84
Education, yrs (SD)	16.20 (2.71)	16.61 (2.57)	15.96 (2.84)	16.08 (2.56)	15.96 (2.74)	*X*^2^(3) = 17.96, *P* < 0.001*	16.50 (2.68)	16.11 (2.57)	W = 272, *P* = 0.47	15.89 (2.77)	16.02 (2.71)	t = 1.54 ×10^4^, *P* = 0.64
**Sex**												
Male	766	219	272	64	210	--	24	40	--	114	96	--
Female	675	291	186	52	147	--	13	39	--	67	80	--
*APOE* **status, n individuals**												
ε4 carriers	581	138	188	41	214	*X*^2^(9) = 111.3, *P* < 0.001*	10	31	X^2^(2) = 1.84, *P* = 0.61	116	98	*X*^2^(2) = 0.45, *P* = 0.80
ε2 carriers	121	66	35	8	12		3	5		5	7	
ε24 genotype	29	8	8	2	11		1	1		6	5	
ε33 genotype	710	298	227	65	120		23	42		54	66	
**Study measures, mean (SD)**												
CDR global	0.41 (0.45)	0.38 (0.45)	0.40 (0.44)	0.46 (0.50)	0.45 (0.44)	*X*^2^(3) = 7.23, *P* = 0.06	0.50 (0.24)	0.46 (0.51)	W = 373, *P* = 0.43	0.45 (0.43)	0.44 (0.47)	W = 1.64 × 10^4^, *P* = 0.50
ADAS-cog	16.25 (8.63)	11.35 (4.66)	18.42 (8.41)	16.21 (6.51)	20.49 (10.35)	*X*^2^(3) = 303.14, *P* < 0.001*	15.36 (7.68)	16.23 (6.57)	W = 220, *P* = 0.96	20.41 (10.87)	20.57 (9.81)	W = 1.54 × 10^4^, *P* = 0.59
GDS-12, mean (SD)	4.49 (1.06)	4.11 (0.88)	4.72 (1.11)	4.37 (0.91)	4.77 (1.06)	*X*^2^(3) = 154.92, *P* < 0.001*	4.33 (0.82)	4.38 (0.91)	W = 276, *P* = 0.39	4.79 (1.07)	4.75 (1.09)	W = 1.67 × 10^4^, *P* = 0.39
**Medications, n individuals**												
Total	290	146	27	54	63	*X*^2^(9) = 3.72, *P* = 0.93	37	17	*X*^2^(2) = 6.77, *P* = 0.03*	32	31	*X*^2^(2) = 2.24, *P* = 0.33
SSRI	163	77	14	33	39		18	15		23	16	
SNRI	44	24	3	6	11		6	0		4	7	
Atyad	93	47	12	16	18		13	3		8	10	

(*) Indicate Statistically Significant Contrasts. Note: Some Individuals May be on Multiple Types of Medications.

### Cognitively Normal to Mild Cognitive Impairment

#### Apathy and *APOE*

**Figure 2.**
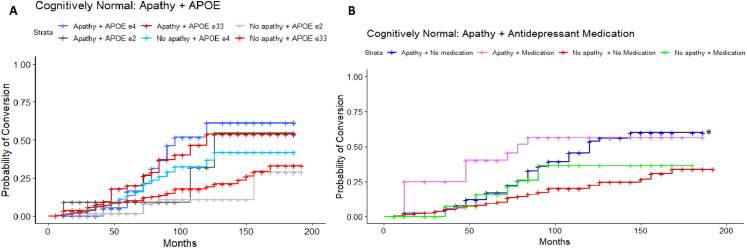
Estimated conversion functions for CN participants according to apathy status and A) *APOE* ε4 genotype. (B) Antidepressant medication status: those with apathy and not on medication (blue, starred) show a faster rate of conversion than those without apathy and not on antidepressants (red, reference category).

Results of the adjusted Cox proportional-hazards model in the CN to MCI cohort (LRT: λ(11) = 86.26, *P* < 0.001) revealed a significant 2-fold increase in risk of conversion in *APOE* ε4 carriers who endorsed apathy compared to the reference group, *APOE* ε33 individuals without apathy (HR = 2.05, 95%CI = 1.04-4.02, *P* = 0.04; Figure 2(A)). Individuals with an *APOE* ε33 genotype and who did endorse apathy had an 84% increased risk of conversion compared to their non-apathetic counterparts (HR = 1.84, 95%CI = 1.08-3.13, *P* = 0.02). An increased risk of conversion for individuals *APOE* ε2 carriers with apathy (HR = 2.08, 95%CI = 0.62-7.03, *P* = 0.24), and attenuated risk for *APOE* ε2 carriers without apathy (HR = 0.57, 95%CI = 0.22-1.44, *P* = 0.23) was found to be non-significant. A higher ADAS-Cog score significantly increased risk of conversion by 9% (HR = 1.09, 95%CI = 1.06-1.11, *P* < 0.001). Post-hoc pairwise comparisons of estimated marginal means, using Tukey correction for multiple comparisons, revealed significant differences in hazards between apathetic ε4 carriers and non-apathetic ε33 individuals (*Q* = 0.72, *P* = 0.047), and apathetic vs non-apathetic ε33 individuals (*Q* = 0.61, *P* = 0.049). However, no statistically significant difference was found between apathetic and non-apathetic ε4 carriers (*Q* = 0.33, *P* = 0.80) or ε2 carriers (*Q* = 1.30, *P* = 0.24). After applying Bonferroni corrections for multiple comparisons, ADAS-cog score was the only significant predictor of conversion (Table S1). A sensitivity model excluding CDR global scores was consistent with findings from the main analysis.

#### Apathy and Antidepressant Medication

A separate adjusted Cox proportional-hazards model was used to assess the combined effects of apathy and antidepressant medication on conversion, to delineate whether apathy unrelated to use of anti-depressants was associated with an increased risk of conversion. The reference group comprised CN participants without apathy and not on any antidepressant. The model was statistically significant (LRT: λ(9) = 82.05, *P* < 0.001), and demonstrated that participants who endorsed apathy but were not taking an antidepressant medication (SSRI, SNRI or AYTADs) had a 78% increased risk of conversion to MCI (HR = 1.78, 95%CI = 1.16-2.75, *P* = 0.009; Figure 2(B)). Again, a higher ADAS-Cog score significantly increased risk of conversion by 9% (HR = 1.09, 95%CI = 1.07-1.11, *P* < 0.001). Post-hoc pairwise comparisons revealed a significant difference between individuals with apathy not on medication and those without apathy not on medication (*Q* = 0.58, *P* = 0.045). However, no significant difference was found between those with apathy on medication vs off medication (*Q* = 0.16, *P* = 0.98). All predictors survived Bonferroni correction. A sensitivity model excluding CDR global scores was consistent with findings from the main analysis.

#### Apathy, *APOE*, and Antidepressant Medication

**Figure 3.**
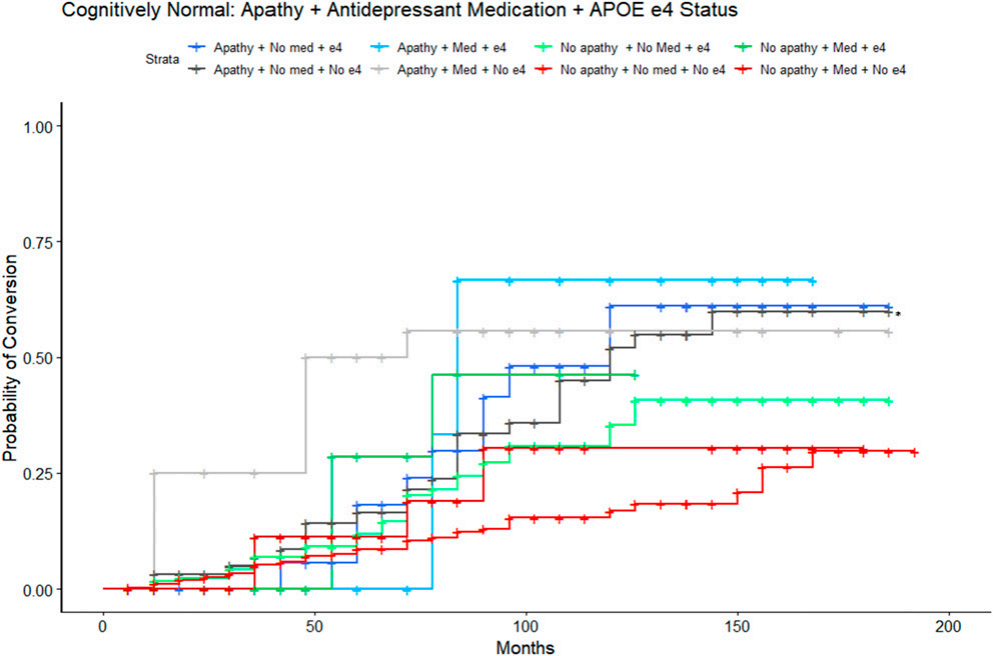
Estimated conversion functions for MCI participants: Depression medication, Apathy, and *APOE ε*4 status. CN participants with apathy, not on an antidepressant, and without an *APOE ε*4 allele (dark grey, starred) convert to MCI at a faster rate compared to those without apathy, not taking antidepressant medication, and without the possession of an *APOE ε*4 allele (red, reference category).

To characterize the potential interactions of apathy, *APOE* ε4 status and depression medications on conversion risk, a separate adjusted Cox proportional-hazards analysis was conducted with all 3 variables. The reference group was CN individuals without apathy, not on an antidepressant medication, and without an *APOE* ε4 allele. The adjusted model was significant (LRT: λ(13) = 86.95, *P* < 0.001) and revealed a 2-fold increase in risk of conversion for those who were apathetic, not on an antidepressant medication and with the possession of *APOE* ε4 allele (HR = 2.12, 95%CI = 1.02-4.423, *P* = 0.044; Figure 3) or without an *APOE* ε4 allele (HR = 2.08, 95%CI = 1.23-3.52, *P* = 0.006). A 3-fold increase in risk of conversion was revealed for individuals without apathy, on an antidepressant medication, and with the possession of an *APOE* ε4 allele (HR = 3.50, 95%CI = 1.10-11.48, *P* = 0.038). Post-hoc comparisons, with Tukey correction, revealed no statistically significant pairwise differences. After Bonferroni correction, the combination of apathy, no *APOE* ε4 allele, and no antidepressant medication remained statistically significant, as did ADAS-Cog score. A sensitivity model excluding CDR global scores was consistent with findings from the main analysis.

### Mild Cognitive Impairment to Alzheimer’s Disease Dementia

#### Apathy and *APOE*

**Figure 4.**
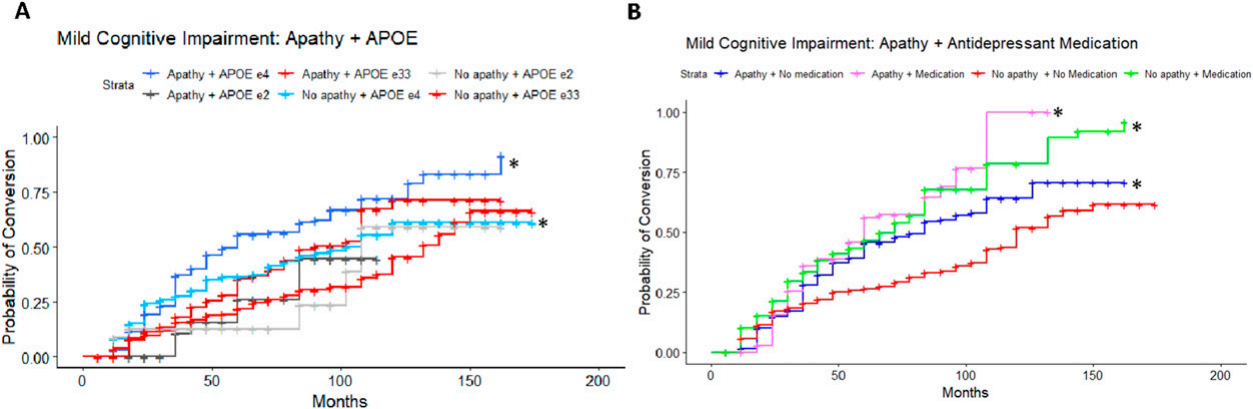
Estimated conversion functions for MCI participants. (A) Both apathetic (navy blue, starred) and non-apathetic (blue, starred) *APOE ε*4 carriers convert to ADD at a faster rate compared to the *APOE ε*33 non-apathetic reference category (red). (B) Participants with apathy and not on a medication (blue, starred) convert at a faster rate compared to those without apathy not on medication. Participants with apathy taking an antidepressant medication (pink, starred) and those with no apathy and on an antidepressant medication (green, starred) convert at a faster rate compared to the reference category.

In the MCI to ADD cohort, results of the adjusted Cox proportional-hazards model (LRT: λ(11) = 70.32, *P* < 0.001) revealed a 2-fold increase in conversion risk in MCI *APOE* ε4 carriers who endorsed apathy (HR = 2.64, 95%CI = 1.93-3.62, *P* < 0.001; Figure 4(A)) in comparison to the reference category of non-apathetic ε33 carriers. *APOE* ε4 carriers without apathy had a 72% increase in risk of conversion (HR = 1.72, 95%CI = 1.24-2.38, *P* = 0.001). Additionally, individuals with apathy and an *APOE* ε33 genotype had a 52% increased risk of conversion (HR = 1.52, 95%CI = 1.06-2.19). A higher ADAS-Cog score was found to increase risk of conversion by 2% (HR = 1.02, 95%CI = 1.01-1.03, *P* < 0.001). Post-hoc pairwise comparisons, with Tukey corrections, revealed significant differences between the following pairs: Apathetic ε4 carriers and apathetic ε33 individuals (*Q* = 0.55, *P* = 0.010), apathetic and non-apathetic ε4 carriers (*Q* = 0.43, *P* = 0.025), apathetic ε4 carriers and non-apathetic ε33 individuals (*Q* = 0.97, *P* < 0.0001), non-apathetic ε4 carriers and ε33 individuals (*Q* = 0.54, *P* = 0.015). Overall, apathetic ε4 carriers demonstrated significantly higher risk of conversion compared to non-apathetic ε4 carriers. Risk associated with apathetic participants with an ε33 genotype did not survive Bonferroni correction. A sensitivity model excluding CDR global scores was consistent with findings from the main analysis.

#### Apathy and Antidepressant Medication

A separate adjusted model exploring antidepressant medication use (SSRI, SNRI or AYTADs) and apathy was statistically significant (LRT: λ(9) = 59.32, *P* < 0.001). The reference group was participants with MCI who did not endorse apathy and were not on an antidepressant. Participants who were apathetic and not on an antidepressant medication demonstrated a 63% increased risk of conversion (HR = 1.63, 95%CI = 1.29 = 2.05, *P* < 0.001). A 2-fold increased risk of conversion to ADD was observed in participants who were taking antidepressant medication whether apathetic (HR = 2.54, 95%CI = 1.68-3.85, *P* < 0.001; Figure 4(B)) or non-apathetic (HR = 2.28, 95%CI = 1.51-3.44, *P* < 0.001). Post-hoc pairwise comparisons revealed significantly higher risk associated with apathetic vs non-apathetic individuals not on medication (*Q* = 0.49, *P* < 0.001), higher risk in apathetic individuals on medication compared to non-apathetic individuals not on medication (*Q* = 0.93, *P* < 0.001), and lastly, higher risk of conversion for non-apathetic individuals on medication vs not on medication (*Q* = 0.82, *P* < 0.001). All predictors survived Bonferroni correction. A sensitivity model excluding CDR global scores was consistent with findings from the main analysis.

#### Apathy, *APOE*, and Antidepressant Medication

**Figure 5.**
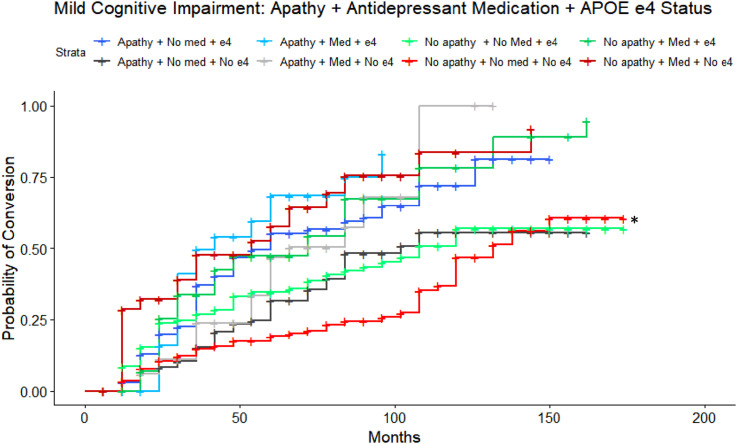
Estimated conversion functions for MCI participants: Depression medication, Apathy, and *APOE* ε4 status. All groups convert from MCI to AD at a faster rate than the reference group of those who do not endorse apathy, do not take depression medication, and without an *APOE* ε4 allele (red, reference group, starred).

Lastly, a sensitivity analysis to identify potential interactions amongst apathy, *APOE* ε4 status and depression medications on conversion rate was conducted. The model was significant (LRT: λ(13) = 89.65, *P* < 0.001), where individuals with MCI, no apathy, not on antidepressant medications, and without an *APOE* ε4 allele were used as the reference group. Overall, results demonstrated significant three-factor interactions in accordance with the main findings of the two-factor Apathy + *APOE* ε4 and Apathy + Antidepressant analyses. Most notably, the main finding of greater risk of conversion with apathy and with antidepressant use was present across both *APOE* ε4 carriers (HR = 4.24, 95%CI = 2.38-7.53, *P* < 0.001) and non-carriers (HR = 2.82, 95%CI = 1.51-5.28, *P* = 0.001, Figure 5). All predictors survived Bonferroni correction. Post-hoc pairwise comparisons, with Tukey corrections, revealed significant differences, as detailed in Table 2. A sensitivity model excluding CDR global scores was consistent with findings from the main analysis.

**Table 2.** MCI to AD: Statistically Significant Pairwise Comparisons for Apathy + *APOE* + Antidepressant Interaction.

	Apathy + No med + ε4	Apathy + Med + ε4	Apathy + Med + No ε4	No apathy + No med + ε4	No apathy + Med + ε4	No apathy + Med + No ε4
Apathy + No med + No ε4	Q = 0.72, *P* = 0.001	Q = 1.09, *P* = 0.007	--	--	--	--
No apathy + No med + ε4	Q = 0.49, *P* = 0.017	Q = 0.86, *P* = 0.047	--	--	--	--
No apathy + No med + No ε4	Q = 1.08, *P* < 0.0001	Q = 1.44, *P* < 0.0001	Q = 1.04, *P* = 0.026	Q = 0.58, *P* = 0.015	Q = 1.11, *P* = 0.004	Q = 1.18, *P* = 0.004

## Discussion

This study aimed to identify the associations of apathy, *APOE* ε4 allele status, and antidepressant medication use with progression from CN to MCI, and from MCI to ADD. Our results support previous findings on apathy and *APOE* ε4 allele status and provide novel insights on patterns of apathy and conversion related to antidepressant use.

We first examined whether apathy interacts with the genetic risk factor for conversion, *APOE* ε4 allele possession. In both cohorts, CN to MCI and MCI to ADD, apathetic *APOE* ε4 carriers indeed demonstrated the highest risk of conversion, with a relatively greater effect (hazards ratio) of conversion in the MCI to ADD group. In the CN to MCI group, risk for conversion in apathetic *APOE* ε4 carriers did not survive Bonferroni corrections. These findings support the role of apathy-*APOE* ɛ4 interactions in conversion to ADD from MCI.^8,21^ Our results may demonstrate an increase of risk of conversion to MCI associated with apathy and the *APOE* ε4 allele in *cognitively normal* individuals, and to a lesser extent, individuals with apathy and an *APOE* ε33 genotype. This finding differs from a recent investigation in CN individuals, where apathetic *APOE* ε3 carriers had a higher risk of developing dementia than apathetic *APOE* ε4 carriers^22^; the results were, however, not specific to ADD and included individuals who progressed to frontotemporal dementia, Lewy body dementia, and vascular dementia, as well. The authors proposed that the relative risk of conversion to dementia is lower in *APOE* ε4 carriers, compared to *APOE* ε3, because progression is likely driven by additional pathological pathways related to the ε4 allele, and unrelated to apathy. Our findings, therefore, introduce a plausible novel link between apathy + *APOE* ε4 and progression from CN to MCI, specific to individuals with amnestic MCI (ADD prodrome).

Next, we investigated whether antidepressant use could be moderating the association between apathy and conversion. In both cohorts, there was no significant difference in risk of conversion in our contrast of interest between apathetic individuals taking antidepressants vs apathetic individuals not taking antidepressants. Compared to the reference group (no apathy and not taking antidepressant), results of the CN to MCI cohort revealed no combined association of apathy and medication use on conversion. Conversely, in the MCI to ADD group, apathy and antidepressant use yielded an over 2-fold heightened risk of conversion compared to the reference group, even when adjusting for depression severity. These findings did not align with our expectation of similar risk predictors along the CN to MCI to ADD continuum, as antidepressant use was not a significant factor in CN to MCI conversion. This may be, in part, explained by the relative efficacy of antidepressants for depression in CN persons vs those with MCI.^23^ In a longitudinal examination of late-life depression and conversion to MCI and AD, cognitively normal adults with depression that improved over time had a decreased risk of conversion to MCI compared to those who endorsed depression throughout the study visits.^23^ Although antidepressant use was not a significant moderator of the observed effect, the study did not account for antidepressant dose and type effects. Our findings, therefore, may point to a more robust association between general antidepressant use and apathy in individuals with MCI converting to ADD than CN individuals converting to MCI.

Lastly, we examined whether the combined effects of apathy and antidepressant use were moderated by *APOE* status. In the CN to MCI group, participants with apathy, not on an antidepressant, and no *APOE* ε4 allele, converted 2-times faster than their non-apathetic counterparts who were not on an antidepressant and non-carriers (reference group). These findings align with our initial 2 analyses, as described above. Interestingly, non-apathetic *APOE* ε4 carriers on an antidepressant medication showed over a 3-fold increased risk of conversion from CN to MCI, compared to the reference group. Although this result did not survive Bonferroni correction, it can be interpreted within the context of a double-blind randomized controlled trial investigating the efficacy of antidepressants in CN adults, aged 65 and above, with major depressive disorder; the study showed that *APOE* ε4 carriers responded rapidly to mirtazapine, whereas paroxetine-treated patients were slow to respond.^24^ Likewise, a 6-year longitudinal study in community-dwelling older adults found that the effect of depressive symptoms on cognitive decline was exacerbated for *APOE* ε4 carriers compared to non-carriers.^25^ Accordingly, in the current study, “antidepressant use” in CN *APOE* ε4 carriers is associated with increased risk of conversion to MCI, possibly as a consequence of depression severity, or depression unresponsive to treatment; in this sense, antidepressant use may be interpreted as a proxy for depression severity or depression history.

In the MCI to ADD group, apathetic *APOE* ε4 carriers on antidepressants demonstrated the highest risk of conversion, with an over 4-fold increased risk. Of interest, the combination of apathy and antidepressant use was not associated with an increase in risk compared to antidepressant use alone (without apathy). These findings suggest that antidepressant use and *APOE* ε4 status may be stronger predictors of conversion to ADD than apathy endorsement, but the combined risk associated with all 3 of these predictors is strongest. A potential reason for why antidepressant use is a predictor of conversion is that it may be a *proxy* for apathy or depression severity. Previous research describes antidepressant-induced apathy,^14^ which may cause or enhance apathy or apathy severity in our study. Alternatively, antidepressant use may indicate the presence of untreated depression. Indeed, though relatively few clinical trials of antidepressants for depression in dementia have been conducted to date, the largest studies have shown limited therapeutic efficacy of antidepressant use in this population,^26-28^ demonstrating that depression is likely present and resistant to treatment in our MCI to ADD group. Some studies have shown that depressive symptoms can increase risk of dementia in individuals with MCI.^29^ However, few studies have examined how depression is phenomenologically different in dementia compared to cognitively normal adults.^30^ Although apathy is increasingly recognized as an independent and multidimensional clinical construct, symptoms of apathy are present in individuals with depression; this makes it difficult to parse out phenomena specific to apathy from those of depression associated with apathy. Previous studies of depressive symptom profiles in dementia long-term care facilities reveal that motivational symptoms, such as apathy, and irritability are the most prevalent.^31,32^ As such, it may be the case that antidepressant use in pre-dementia and dementia is prescribed to treat “depressive” symptoms more consistent with apathy and cognitive profiles. As there is no gold-standard biomarker to discriminate apathy from depression, in the current study, the GDS-12 was used to quantify symptoms of depression excluding items related to apathy symptoms. The GDS-12 was not a significant predictor of increased risk of conversion, supporting this interpretation of apathy-predominant symptoms in our cohort, however, this approach does not rule out possible contributions from depression to the apathy-only group.

Of the 3 antidepressant classes included in the current study (SSRIs, SNRIs, AYTADs), most participants reported taking an SSRI. Previous studies describe a dose-dependent apathy specific to SSRI use.^14,33,34^ 1 study found that, although SSRIs generally induced symptoms of apathy in a cohort of geriatric patients, fluoxetine and paroxetine use had the highest rates of apathy, followed by citalopram, then sertraline, and finally escitalopram.^14^ The authors proposed that different SSRIs, at variable doses, can have a dual impact on pertinent neurotransmitter systems, via serotonergic and midbrain dopaminergic modulation of systems projecting to the prefrontal cortex. As a result, a hypodopaminergic state may lead to increased symptoms of apathy, depending on the dose and action of the SSRI.^35^ Similarly, SNRIs are dually serotonin and norepinephrine reuptake inhibitors. Norepinephrine is another neurotransmitter implicated in apathy symptomatology.^2,35^ However, it could be the case that SNRI dual-action properties abate symptoms of apathy. One recent case study reported 2 patients who experienced apathy induced by low-dose venlafaxine that was later completely reversed by administration of a higher dose.^36^ The authors speculated that higher norepinephrine reuptake activity, compared to serotonin reuptake activity, occurred at higher doses of venlafaxine. These converging lines of research provide some crucial insight into our findings. SSRI use was most common in our participants, and few participants endorsed taking SNRIs, and even fewer took AYTADs; it is likely that these proportions of medication use either induced or exacerbated apathy or did not help abate pre-existing symptoms of apathy. However, our results do not justify a causal link between antidepressant use and conversion.^37^

There were several limitations to this study. One limitation of the present study is the small sample size. As a consequence, a relatively small number of individuals taking antidepressants in the various classes (ie, SSRIs, SNRIs, and AYTADs) were included in the study, which did not support modeling of drug-specific effects and dose-dependent effects. An inability to account for dose-related effects was a significant limitation in this study, as previous work supports dose-dependent induced apathy by antidepressant use.^14,33,34^ Similarly, given the smaller sample, other medications in our group of poly-morbid and poly-medicated participants, could not be considered in the current analysis Table S2 provides some insight into the concomitant medications in our sample. Chi-squared tests of independence were used to determine whether significant imbalances between groups exist in the number of participants on cognition- or mood-altering drugs. Importantly, in CN with and without apathy, the number and proportion of participants taking anti-psychotics, benzodiazepines, gaba-ergic medications and β-blockers was similar in absolute number and proportion to those converting from CN to MCI with and without apathy. No statistically significant imbalances were seen in the number of MCI to AD converters vs MCI non-converters. These findings suggest that a drug-specific effect of these medications may not play a major role in conversion in our sample. This is perhaps due to certain classes of medications, such as cholinesterase inhibitors, being indicated following the onset of MCI or AD diagnosis.

Further, while we accounted for depression in our adjusted models to separate associations of apathy from depression, the GDS-12 may be subject to self-report biases and imperfectly reflect the degree of depression related physiologic changes in individuals. Given the binary measure of apathy afforded by the NPI-Q, we were also unable to account for apathy severity. Importantly, we could not determine whether apathy was endorsed before or after antidepressant treatment began; as such, we do not know whether any of our participants experienced antidepressant-induced apathy. To conserve power in this study, *APOE* ε4 homozygous and heterozygous carriers were combined. As 2 *APOE* ε4 alleles are associated with greater risk for ADD than 1 allele, parsing out dose-related effects of *APOE* ε4 on apathy and progression risk in larger samples is warranted for future work. In the absence of data from randomized clinical trials, future studies, with larger samples, should aim to include antidepressant class- and dose-stratified variables.

In summary, we found combined effects of apathy and *APOE* ε4 status were associated with increased risk of conversion from CN to MCI and MCI to ADD. Antidepressant use, potentially serving as a proxy for depression or apathy severity, was associated with progression from MCI to ADD, and potentially progression from CN to MCI in non-apathetic *APOE* ε4 carriers. Although these findings are rudimentary and represent associations, they are consistent with recent large observational studies in other cohorts which have found correlations between SSRI use and dose with faster cognitive decline in persons with dementia.^38^ Practically, even as observational associations, they may inform future clinical considerations around prescribing antidepressants in individuals at-risk for dementia. For instance, routine inclusion of apathy questionnaires and clinical ascertainment of apathy in the absence of depression may reduce the prescription of SSRIs for isolated apathy, which is known not to respond to this treatment. However, future studies, and ideally randomized controlled trials of antidepressants, including nuanced measures of apathy and depression symptoms and antidepressant dose, are warranted to better study potential causal relationships between antidepressant use and progression to MCI and ADD.

## Supplemental Material

Supplemental Material - Conversion to Mild Cognitive Impairment and Alzheimer’s Disease Dementia Related to Apathy, *APOE* Genotype and Antidepressant Use Supplemental Material for Conversion to Mild Cognitive Impairment and Alzheimer’s Disease Dementia Related to Apathy, *APOE* Genotype and Antidepressant Use by Rubina Malik, Miguel Restrepo Martinez, Isis So, Elizabeth Finger, and for the Alzheimer’s Disease Neuroimaging Initiative in Journal of Geriatric Psychiatry and Neurology

## Data Availability

All data generated or analyzed during this study are available upon request to the corresponding author.*
