# Supplementary material for: Conversion to Mild Cognitive Impairment and Alzheimer’s Disease Dementia Related to Apathy, APOE Genotype and Antidepressant Use
Source: J Geriatr Psychiatry Neurol. 2025 Apr 14;38(6):484–97. doi: 10.1177/08919887251335002 (PMC12433532; doi:10.1177/08919887251335002)
Supplement: Supplemental Material - Conversion to Mild Cognitive Impairment and Alzheimer’s Disease Dementia Related to Apathy, APOE Genotype and Antidepressant Use [file sj-pdf-1-jgp-10.1177_08919887251335002.pdf]

Cognitively Normal to Mild Cognitive Impairment (n=626)				
**Model**	**Predictors**	**Coeff (HR)**	***p* Value**	**95% CI**
Apathy + *APOE* ε4 Status	Apathy + ε4	2.04632	0.0377*	1.04-4.02
	No Apathy + ε4	1.46772	0.1081	0.92-2.34
	Apathy + ε2	2.08399	0.2364	0.62-7.03
	No Apathy + ε2	0.56739	0.2326	0.22-1.44
	Apathy + ε33	1.84216	0.0242*	1.08-3.13
	No Apathy + ε33 (*ref*)	--	--	--
	CDR Global	1.11499	0.5800	0.76-1.64
	Age	1.01390	0.3029	0.99-1.04
	Sex	0.82960	0.3414	0.56-1.22
	Education (yrs)	0.94947	0.1532	0.88-1.02
	GDS-12	1.19706	0.0854	0.98-1.47
	ADAS-Cog	1.08781	<0.0001**	1.06-1.11
	**LRT**	**86.26 on 11df, *p*<0.0001**		
Apathy + Antidepressant Medication	Apathy + Med	2.09967	0.11579	0.83-5.29
	Apathy + No Med	1.78309	0.00901**	1.16-2.76
	No Apathy + Med	1.57049	0.26520	0.71-3.47
	No Apathy + No Med (*ref*)	--	--	--
	CDR Global	1.12012	0.56580	0.76-1.65
	Age	1.01356	0.30907	0.99-1.04
	Sex	0.81935	0.32047	0.55-1.21
	Education (yrs)	0.94680	0.13064	0.88-1.02
	GDS-12	1.18583	0.09843	0.97-1.45
	ADAS-Cog	1.08988	<0.0001**	1.07-1.11
	**LRT**	**82.05 on 9df, *p*<0.0001**		
Apathy + *APOE* ε4 Status + Antidepressant Medication	Apathy + ε4 + Med	3.49048	0.0905	0.82-14.84
	Apathy + No ε4 + Med	2.07288	0.2306	0.63-6.83
	Apathy + No ε4 + No Med	2.08294	0.0061**	1.23-3.52
	Apathy + ε4 + No Med	2.12279	0.0440*	1.02-4.42
	No Apathy + ε4 + Med	3.50285	0.0384*	1.07-11.48
	No Apathy + No ε4 + Med	1.37648	0.5489	0.48-3.91
	No Apathy + ε4 + No Med	1.56397	0.0693	0.97-2.53
	No Apathy + No ε4 + No Med (*ref*)	--	--	--
	CDR Global	1.12274	0.5590	0.76-1.66
	Age	1.01470	0.2724	0.99-1.04
	Sex	0.80746	0.2852	0.55-1.20
	Education (yrs)	0.94683	0.1310	0.88-1.02
	GDS-12	1.19096	0.0935	0.97-1.46
	ADAS-Cog	1.08712	<0.001**	1.06-1.11
	**LRT**	**86.95 on 13df, *p*<0.0001**		
**Mild Cognitive Impairment to Alzheimer's Disease (n=815)**				
**Model**	**Predictor**	**Coeff (HR)**	***p* Value**	**95% CI**
Apathy + *APOE* ε4 Status	Apathy + ε4	2.642244	<0.001**	1.93-3.62
	No Apathy + ε4	1.721541	0.00107**	1.24-2.38
	Apathy + ε2	0.945797	0.90499	0.38-2.36
	No Apathy + ε2	0.839651	0.68384	0.36-1.95
	Apathy + ε33	1.519424	0.02431*	1.06-2.19
	No Apathy + ε33 (*ref*)	--	--	--
	CDR Global	0.954893	0.69291	0.76-1.20
	Age	1.007283	0.29510	0.99-1.02
	Sex	1.112694	0.34481	0.89-1.39
	Education (yrs)	1.024763	0.20995	0.99-1.07
	GDS-12	1.062983	0.18391	0.97-1.16
	ADAS-Cog	1.021178	<0.001**	1.01-1.03
	**LRT**	**70.32 on 11df, *p*<0.0001**		
Apathy + Antidepressant Medication	Apathy + Med	2.541724	<0.001**	1.68-3.85
	Apathy + No Med	1.628599	<0.001**	1.29-2.05
	No Apathy + Med	2.280243	<0.001**	1.51-3.44

	No Apathy + No Med (*ref*)	--	--	--
	CDR Global	0.965463	0.763	0.77-1.21
	Age	1.004825	0.472	0.99-1.02
	Sex	1.057528	0.621	0.85-1.32
	Education (yrs)	1.024428	0.214	0.99-1.06
	GDS-12	1.064747	0.165	0.97-1.16
	ADAS-Cog	1.020364	<0.001**	1.01-1.03
	**LRT**	**59.32 on 9df, *p*<0.0001**		
Apathy + *APOE* ε4 Status + Antidepressant Medication	Apathy + ε4 + Med	4.237374	<0.001**	2.38-7.53
	Apathy + No ε4 + Med	2.820313	0.001173**	1.51-5.28
	Apathy + No ε4 + No Med	1.422758	0.076781	0.96-2.10
	Apathy + ε4 + No Med	2.933610	<0.0001**	2.12-4.06
	No Apathy + ε4 + Med	3.034492	0.000152**	1.71-5.39
	No Apathy + No ε4 + Med	3.257104	0.000143**	1.77-5.99
	No Apathy + ε4 + No Med	1.789115	0.000628**	1.28-2.50
	No Apathy + No ε4 + No Med (*ref*)	--	--	--
	CDR Global	0.964857	0.760640	0.77-1.22
	Age	1.010664	0.126199	0.10-1.02
	Sex	1.086289	0.468868	0.87-1.36
	Education (yrs)	1.028865	0.144060	0.99-1.07
	GDS-12	1.062697	0.188466	0.97-1.16
	ADAS-Cog	1.020418	<0.0001**	1.01-1.03
	**LRT**	**89.65 on 13df, *p*<0.001**		

Table S1. Cox proportional Hazards Analyses Results for CN to MCI and MCI to AD.
(*) indicates significance with α= 0.05.
(**) indicates significance after Bonferroni correction for multiple comparisons with α=0.017.

	**CN Stable**	**CN Stable + Apathy**	**CN to MCI**	**CN to MCI + Apathy**	**MCI Stable**	**MCI Stable + Apathy**	**MCI to AD**	**MCI to AD + Apathy**
**Acetylcholinesterase Inhibitors**<br>*Razadyne*<br>*Aricept*<br>*Reminyl*<br>*Rivastigmine*	6	0	8	0	87	78	113	129
**Contrasts:**	$\chi2(1)=0.00$, $p=1.00$				$\chi2(1)=1.20$, $p=0.27$			
**Atypical Antipsychotics**<br>*Seroquel*<br>*Risperdal*<br>*Haloperidol*<br>*Abilify*<br>*Zyprexa*	4	0	1	0	7	3	3	5
**Contrasts:**	$\chi2(1)=3.66\times10^{-32}$, $p=1.00$				$\chi2(1)=0.81$, $p=0.37$			
**Benzodiazepines**<br>*Chlordiazepoxide*<br>*Oxazepam*<br>*Diazepam*<br>*Clonazepam*<br>*Lorazepam*<br>*Clorazepate*<br>*Alprazolam*	37	3	7	2	26	18	6	11
**Contrasts:**	$\chi2(1)=0.50$, $p=0.48$				$\chi2(1)=1.91$, $p=0.17$			
**GABAergic Modulators**<br>*Memantine*<br>*Gabapentin*<br>*Lamotrigine*<br>*Valproate*	37	2	6	0	43	43	50	67

**Contrasts:**	$\chi2(1)=3.93x10^{-30}$, p=1.00				$\chi2(1)=0.78$, p=0.38			
**Beta-Blockers**<br>*Atenolol*<br>*Bisoprolol*<br>*Metoprolol*<br>*Timolol*<br>*Labatolol*<br>*Propanolol*	98	7	19	0	65	48	30	32
**Contrasts:**	$\chi2(1)=0.38$, p=0.54				$\chi2(1)=1.00$, p=0.32			

Table S2. Number of participants on concomitant medications by drug type and participant diagnosis group. All converters included in the table were on medications prior to the conversion event.
